# The potential role of GLUT4 transporters and insulin receptors in the hypoglycaemic activity of *Ficus lutea* acetone leaf extract

**DOI:** 10.1186/1472-6882-14-269

**Published:** 2014-07-28

**Authors:** Oyinlola O Olaokun, Lyndy J McGaw, Maurice D Awouafack, Jacobus N Eloff, Vinny Naidoo

**Affiliations:** Phytomedicine Programme, Department of Paraclinical Sciences, University of Pretoria, Private Bag X04, Onderstepoort, Pretoria, 0110 South Africa; Biomedical Research Centre, Faculty of Veterinary Sciences, University of Pretoria, Onderstepoort, 0110 Pretoria, South Africa; Federal Institute of Industrial Research Oshodi (FIIRO), Lagos, Nigeria; Department of Chemistry, University of Dschang, Dschang, Cameroon

**Keywords:** African *Ficus* species, *Ficus lutea*, Diabetes, Glucose uptake, Insulin secretion

## Abstract

**Background:**

Some *Ficus* species have been used in traditional African medicine in the treatment of diabetes. The antidiabetic potential of certain species has been confirmed *in vivo* but the mechanism of activity remains uncertain.

The aim was to investigate the hypoglycaemic potential of ten *Ficus* species focussing on glucose uptake, insulin secretion and the possible mechanism of hypoglycaemic activity.

**Methods:**

The dried and ground leaves of ten *Ficus* species were extracted with acetone. The dried acetone extract was reconstituted with DMSO to a concentration of 100 mg/ml which was then serially diluted and used to assay for glucose uptake in muscle, fat and liver cells, and insulin secretion in pancreatic cells.

**Results:**

Only the *F. lutea* extract was able to modulate glucose metabolism. In comparison to insulin in the primary muscle cells, the glucose uptake ability of the extract was 33% as effective. In the hepatoma cell line, the extract was as effective as metformin in decreasing extracellular glucose concentration by approximately 20%. In the pancreatic insulin secretory assay, the extract was 4 times greater in its secretory activity than commercial glibenclamide. With *F. lutea* extract significantly increasing glucose uptake in the primary muscle cells, primary fat cells, C2C12 muscle and H-4-II-E liver cells, the extract may act by increasing the activity of cell surface glucose transporters. When the 3T3-L1 pre-adipocytes were compared to the primary muscle, primary fat and C2C12 cells, the differences in the former’s ability to transport glucose into the cell may be due to the absence of the GLUT4 transporter, which on activation via the insulin receptor decreases extracellular glucose concentrations. Because the pre-adipocytes failed to show any active increase in glucose uptake, the present effect has to be linked to the absence of the GLUT4 transporter.

**Conclusion:**

Only *F. lutea* possessed substantial *in vitro* activity related to glucose metabolism. Based on the effect produced in the various cell types, *F. lutea* also appears to be a partial agonist/antagonist of the insulin cell membrane receptor. While the clinical effectiveness of *F. lutea* is not known, this plant species does possess the ability to modify glucose metabolism.

## Background

In many developing countries, herbal medicines are of vital importance in primary health care [1]. This is supported by literature in behavioural and pharmacological sciences with animals and people using a number of different plants species for the control of disease symptoms and related illnesses [2, 3]. One such disorder is diabetes mellitus (DM) which is a chronic disease characterised by prolonged hyperglycaemia, especially post-prandial, in association with the consumption of diets that promote obesity, due to abnormalities in plasma insulin concentrations.

Under normal physiological conditions, insulin is secreted by the β-cells of the pancreatic Islets of Langerhans in the presence of increased plasma glucose concentrations in a highly controlled manner. This potent anabolic hormone subsequently decreases the plasma glucose concentrations to a specific limit, through the suppression of hepatic glucose production, increased macromolecular synthesis of glycogen and triglycerides, and stimulation of peripheral (skeletal muscle and adipose tissue) glucose uptake [4, 5]. Therefore any defects in the action or production of insulin will lead to physiological hyperglycaemia of DM [5]. Over time, this prolonged chronic hyperglycaemia could result in microvascular and macrovascular damage, causing long term complications such as neuropathy, nephropathy, retinopathy, cardiovascular disease and impaired wound healing that significantly interferes with quality of life and life expectancy [5].

While several types of DM can occur, type I and type II predominate. In type I diabetes, or insulin dependent DM, the body has little or no insulin secretory capacity and depends on exogenous insulin to prevent metabolic disorders and death. In type II diabetes, a non-insulin dependent DM, the body retains some endogenous insulin secretory capability; however, insulin levels are low relative to blood glucose levels and/or there is a measure of insulin resistance. Type II diabetes is the most prevalent form of the disease, accounting for 90-95% of cases globally [6, 7].

While conventional treatments such as sulfonylureas, metformin and thiazolidinedones are effective, they have several limitations, including adverse side effects, secondary failure or the inability to halt further loss of insulin secretory capacity [8]. Newer and cheaper medications are therefore needed. One solution is to use herbal remedies, which appear to be widely used with relatively few documented side effects [9].

Several species of the genus *Ficus* (family Moraceae) are used traditionally in the management of diabetes [10, 11]: *Ficus benghalensis* L. [12], *Ficus carica* L. [13], *Ficus racemosa* L. [14], *Ficus hispida* L. [15], *Ficus microcarpa* L.f. [16], *Ficus religiosa* L. [17], *Ficus thonningii* Blume [18], *Ficus glumosa* Del. [19], *Ficus arnottiana* Miq. [20], *Ficus glomerata* Roxb. [21], *F. sycomorus* L. [22] and *F. deltoidea* Jack [23] *.* Despite their widespread use, the activity and possible mechanism of activity of *Ficus* species is yet to be determined. The aim of this study was therefore to investigate the mechanisms of the antidiabetic activity, if any, of ten selected *Ficus* species.

## Methods

### Reagents and chemicals

Roswell Park Memorial Institute Medium 1640 (RPMI 1640), foetal calf serum (FCS)/ foetal bovine serum (FBS), bovine calf serum (BCS), glutamine, trypsin-EDTA, phosphate buffered saline (PBS), sodium pyruvate, Hanks balanced salt solution (HBSS), glucose oxidase kit (GAGO 20), bovine serum albumin (BSA), Minimum Essential Medium (MEM), Dulbecco’s MEM (DMEM), 3-(4,5-dimethylthiazol-2-yl)-2,5-diphenyl tetrazolium bromide (MTT), 2-[4-(2-hydroxyethyl)piperazin-1-yl]-ethanesulfonic acid (HEPES), collagenase (type 11), penicillin/streptomycin, glibenclamide and D-glucose were purchased from Sigma (South Africa). Gentamicin (Virbac), insulin (Sanofi Aventis), Trypan blue (Fluka), doxorubicin (Pfizer) and Insulin (Rat) ELISA kit (DRG Instruments GmbH, Germany Frauenbergst). Sodium hydrogen carbonate (NaHCO_3_), potassium chloride (KCl), sodium hydrogen phosphate (NaH_2_PO_4_), calcium chloride (CaCl_2_), magnesium sulphate (MgSO_4_), sodium chloride (NaCl), magnesium chloride (MgCl_2_) potassium hydrogen phosphate (KH_2_PO_4_), dimethyl sulphoxide (DMSO), acetone, methanol, hydrogen chloride (HCl), sulphuric acid (H_2_SO_4_) and Whatman No. 1 filter paper were purchased from Merck (South Africa). The absorbance measurements were read using a microtitre plate reader (VERSAmax, Molecular Devices, Labotec).

### Cell lines and primary cell cultures

C2C12 mouse muscle myoblast (CRL-1772), 3T3-L1 mouse pre-adipocytes fibroblast (CL-173), H411E rat hepatoma (CRL-1548), and RIN-m5F rat insulinoma (CRL-11065) were purchased from American Type Culture Collection (ATCC) (Manassas, VA). The primary abdominal muscle and epididymal fat pad were collected opportunistically post-mortem from other animal studies, approved by the Animal Use and Care Committee (AUCC) of the University of Pretoria.

### Plant material

The leaves of the ten *Ficus* species were collected at the Manie van der Schijff Botanical Garden (University of Pretoria), South Africa in February 2009. The names of the plant species are *Ficus capreifolia* Delile, *Ficus cordata* Thunb., *Ficus craterostoma* Warb. ex Mildbr. & Burret, *Ficus glumosa* Delile, *Ficus lutea* Vahl, *Ficus natalensis* Hochst., *Ficus polita* Vahl, *Ficus religiosa* L., *Ficus sycomorus* L., and *Ficus thonningii* Blume. Voucher specimens are placed in the HGWJ Schweickerdt Herbarium of the same institution (for voucher numbers see Olaokun et al. [24]). Leaf material was dried at room temperature, milled to a fine powder (Macsalab mill, Eriez® Bramley) and stored at room temperature in the dark until extracted [25]. Powdered material (2 g) was extracted with acetone (20 ml, Merck technical grade) using a platform shaker (Labotec) at room temperature for 30 min [26]. Extracts were centrifuged at 500 × g for 5 min (Hettich centrifuge) filtered three times (Whatman No. 1 filter paper), prior to drying at room temperature under a stream of cold air. Crude extracts were dissolved in DMSO (100%) to produce stock solutions of 100 mg/ml prior to some of the assays.

### Isolation of compounds

Dried extract (74 g) re-dissolved in 50% acetone in water, was successively and exhaustively partitioned (by liquid-liquid extraction) with hexane, chloroform/dichloromethane, ethyl acetate and n-butanol (in order of increasing polarity) [27]. With the *F. lutea* activity related to glucose utilisation residing in the ethyl acetate fraction, 12 g ethyl acetate fraction was subjected to silica gel column chromatography eluting with increasing polarity of n-hexane (n-hex), ethyl acetate (EtOAc) and methanol (MeOH) mixtures to afford 115 fractions of 500 mL each. Fractions 46–52 eluted with n-hex: EtOAc (70:30) were also subjected to similar silica gel column chromatography as fractions 10–30 followed by preparative TLC to afford a compound of about 21 mg.

#### General experimental procedures

Column chromatography was performed on MN silica gel 60 (0.063-0.2 mm/70–230) mesh. Pre-coated plates of TLC silica gel 60 F_254_ (Merck, Germany) were used for monitoring fractions and spots were detected with UV light (254 and 365 nm) and then sprayed with 3% H_2_SO_4_ followed by heating to 110°C. The structures of compounds were elucidated using ^1^H and ^13^C nuclear magnetic resonance (NMR) with spectra recorded using a Bruker spectrometer at 500 MHz and Variant spectrometer at 400 MHz. Chemical shifts (*δ*) were quoted in parts per million (ppm) from internal standard tetramethylsilane (TMS).

### Glucose uptake in established cell lines

#### Complete growth medium

Frozen vials of cells were revived according to ATCC guidelines. The C2C12 myocytes were maintained in DMEM culture medium supplemented with 10% foetal bovine serum (FBS) and 4 mM glutamine. The 3T3-L1 pre-adipocytes were maintained in DMEM culture medium supplemented with 10% bovine calf serum (BCS) and 4 mM glutamine. The H-4-11-E hepatoma cells were maintained in MEM culture medium supplemented with 10% FBS and 2 mM glutamine, while the RIN-m5F insulinoma cells were maintained in RPMI-1640 with 2 mM glutamine supplemented with 10% FBS, 10 mM HEPES and 1 mM sodium pyruvate.

Glucose uptake was determined by the methods of [28, 29]. The C2C12 myocytes (25 000 cells/ml in DMEM supplemented with 10% FBS), the 3T3-L1 pre-adipocytes (30 000 cells/ml in DMEM supplemented with 10% BCS) and the H-4-11-E hepatoma cells (30 000 cells/ml in MEM supplemented with 10% FBS) seeded (200 μl) into wells of 96-well microtitre plates. After incubation at 37°C in a 5% CO_2_ incubator for 4 days (C2C12 and 3T3-L1) and 2 days (H-4-11-E), the medium in each of the wells was removed and replaced with 100 μl of DMEM supplemented with 0.25% BSA containing plant extracts at concentrations of 15, 31, 63, 125, 250 and 500 μg/ml. The cells were subsequently incubated at 37°C in a 5% CO_2_ incubator for 1 h (C2C12), 1½ h (3T3-L1) and 3 h (H-4-11-E) with the various treatments. Insulin (0.1 - 100 μM) served as the positive control for the C2C12 and 3T3-L1 cells and was incubated for 1 h and 1½ h respectively while metformin and insulin prepared in growth medium (0.1 - 100 μM) were the positive controls for H-4-11-E cells. Cells treated with metformin were incubated for 24 h. The solvent control was 0.5% DMSO. Glucose concentration in the medium was determined by the glucose oxidase method (Sigma GAGO 20 test kit) according to instructions. All experiments were carried out in triplicate and repeated on three separate occasions (n = 9). When a plant extract tended to enhance glucose uptake, the effect was re-tested in the presence of insulin at 1 μM and 10 μM.

### Glucose uptake in primary cell cultures

#### Rat abdominal skeletal muscle

Rat abdominal muscle tissue was prepared using the modified method of Gray and Flatt [30], from 8 week old male Sprague–Dawley rats (n = 12) (170 – 250 g) from the University of Pretoria Biomedical Research Centre. Excised abdominal muscle was washed twice in 10 ml PBS, sectioned into 10-20 g pieces and transferred into 20 ml Krebs-Ringer bicarbonate–bovine serum albumin (KRB-BSA) pH 7.0 (consisting of 118 mM NaCl, 25 mM NaHCO_3_, 1.18 mM MgSO_4_, 1.25 mM CaCl_2_, 5 mM KCl, 1.17 mM KH_2_PO_4_ and 0.2% BSA) before final gentle agitation at 37°C for 20 min in carbogen. Each muscle square was then transferred into micro-titre wells containing 450 μl KRB-BSA supplemented with 2 mM sodium pyruvate, 1 mM glucose and 50 μl of test substance (n = 9) prior to incubation at 30°C for 45 min. Final concentrations were plant extracts (12.5 – 200 μg/ml), insulin (0.1 - 100 μM) and DMSO (1%). Glucose was quantified as above and change in glucose concentration was calculated using the following formula:


#### Rat epididymal adipose cells

Isolated adipocytes were obtained using the method of Rodbell [31] as modified by Martz et al. [32]. The epididymal fat pads (same rats as above) were rinsed in PBS and finely minced and agitated at 37°C for 1 h in DMEM containing 25 mM HEPES, collagenase type 11 (1 mg/ml), and BSA (40 mg/ml). Cells were isolated by first filtration (1000 μm nylon mesh) and subsequent centrifugation at 400 × g for 1 min. The bottom layer was finally aspirated to obtain a cell layer. Isolated cells were washed three times in HEPES buffered Krebs-Ringer solution, pH 7.0, consisting of 20 mM HEPES, 120 mM NaCl, 1.2 mM MgSO_4_, 2.0 mM CaCl_2_, 2.5 mM KCl, 1 mM NaH_2_PO_4_, 1 mM sodium pyruvate and 1% BSA, penicillin (20 units/ml) and streptomycin (20 mg/ml)) prior to final suspension in the same buffer. Adipocyte number and viability were determined by trypan blue exclusion. For assays, primary adipocytes were suspended in medium supplemented with 1 mM D-glucose, in a shaking water bath at 37°C for 20 min. Cell suspension (200 μl) was plated at a density of 1.0 × 10^3^ cells/well in a 96-well microtitre plate with 50 μl of plant extracts (n = 9), to yield final concentrations of plant extract (12.5 – 200 μg/ml), insulin (0.1, 1, 10, 100 μM) and DMSO (1%). Plates were incubated at 37°C in a 5% CO_2_ incubator for 1 h, prior to glucose quantification as above.

### Insulin secretion assay

Insulin secretion by RIN-m5F cells was determined by the method of Persaud *et al.* [33]. The RIN-m5F insulinoma cells (100 000 cells/ml) were suspended in RPMI-1640 supplemented with 10% FBS, 10 mM HEPES and 1 mM sodium pyruvate (200 μl) into 96-well plates. Cells were incubated at 37°C in a 5% CO_2_ incubator for 48 h, after which the medium was exchanged for a glucose–free Krebs-Ringer bicarbonate (KRB) buffer (135 mM NaCl, 3.6 mM KCl, 5 mM NaHCO_3_, 0.5 mM NaH_2_PO_4_, 0.5 mM MgCl_2_ and 1.5 mM CaCl_2_) pH 7.4 supplemented with 1 mg/ml BSA and 10 mM HEPES for a further 2 h of incubation. The medium was subsequently replaced with 100 μl of glucose–free KRB containing plant extract (62.5 - 500 μg/ml), glibenclamide (0.1 - 10 μM) or plain medium, and incubated at 37°C in a 5% CO_2_ for 1 h. The insulin content of supernatants (n = 9) were determined using DRG diagnostic Insulin (Rat) ELISA kit, and the following formula:


To determine toxicity, cells were exposed to the extract or pure compound for 48 h of exposure prior to 3-(4,5-dimethylthiazol-2-yl)-2,5-diphenyl tetrazolium bromide (MTT) assay [34]. In short, once confluent the wells were rinsed with PBS (200 μl) and fresh medium (200 μl) containing 30 μl of MTT (5 mg/ml in PBS) was added to each well and further incubated for 4 h. Subsequently, the medium was aspirated without disturbing the formazan crystals and replaced with 50 μl of DMSO. The absorbance of the coloured formazan was measured at 570 nm after gentle shaking using a microplate reader (VERSAmax). The percentage cell viability was calculated as the absorbance of the treated well divided by the absorbance of the solvent control well. The correlation between percentage cell viability and percentage insulin secretion was carried out.

### Statistical analyses

All experiments were performed in triplicate and repeated on three different occasions to yield nine dose–response curves. Statistical analyses of glucose uptake assays and insulin secretion were done by one-way analysis of variance (ANOVA) and considered to be significantly different at *p* <0.05. When significance was found, location of significance was determined by Bonferroni and Tukey HSD multiple comparison *post hoc* tests. The correlation coefficients (R^2^) between viability of RIN-m5F pancreatic β-cells and insulin secretion were also calculated. All analyses were undertaken in SPSS 20 (IBM). Data are presented as the mean ± standard error of mean (S.E.M.).

## Results

### Glucose uptake activity in C2C12 muscle cells

**Figure 1:**
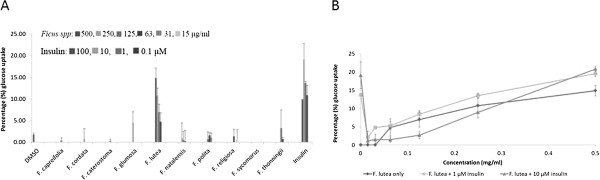
**Glucose uptake in C2C12 muscle cells (A) expressed as percentage of untreated control cells ± standard error of mean, n = 9 exposed to the acetone extracts of the ten** ***Ficus*** **species or insulin and (B) expressed as percentage of untreated control cells ± standard error of mean, (n = 9) exposed to insulin at 1 or 10 μM, in combination with** ***F. lutea*** **at various concentrations.**

The effect of the acetone leaf extracts of the ten *Ficus* species on glucose uptake in C2C12 muscle cells is presented in Figure 1A. Only *F. lutea* extract significantly (*p*<0.001) enhanced glucose uptake in C2C12 muscle cells of 9.9 ± 2.7 μg/ml (14.9 ± 2.3%) at 500 μg/ml, with no activity at concentrations below 63 μg/ml. DMSO (solvent control) had a weak activity of 1.2 ± 0.2 μg/ml (1.8 ± 0.3%), but insulin significantly (*p*<0.001) enhanced glucose uptake of 12.7 ± 3.7 μg/ml (19.1 ± 3.7%) at a 10 μM. With *F. lutea* extract being partially effective on C2C12 cell glucose uptake, the effect of the *F. lutea* in the presence of insulin (1 μM and 10 μM) was evaluated (Figure 1B). Insulin at the two concentrations, significantly (*p*<0.001) increased the glucose uptake in the C2C12 muscle cells. In the presence 1 μM and 10 μM insulin the *F. lutea* extract significantly (*p*<0.05) increased the glucose uptake in the C2C12 muscle cells [13.0 ± 0.6 μg/ml (19.5 ± 0.7%) and 13.9 ± 1.4 μg/ml (20.8 ± 1.6%) respectively] at the highest concentration of 500 μg/ml compared to the *F. lutea* extract alone. A marginal increase was seen for the lower doses of *F. lutea* with insulin present in comparison to its absence. Surprisingly, insulin at both the tested concentrations did not have the maximum effect of 9.12 ± 0.23 μg/ml (13.7 ± 0.36%) and 12.7 ± 3.1 μg/ml (19.1 ± 3.7%), when combined with the lower concentrations of the *F. lutea* extract.

#### Extracellular glucose concentration of H-4-11-E liver cells

**Figure 2:**
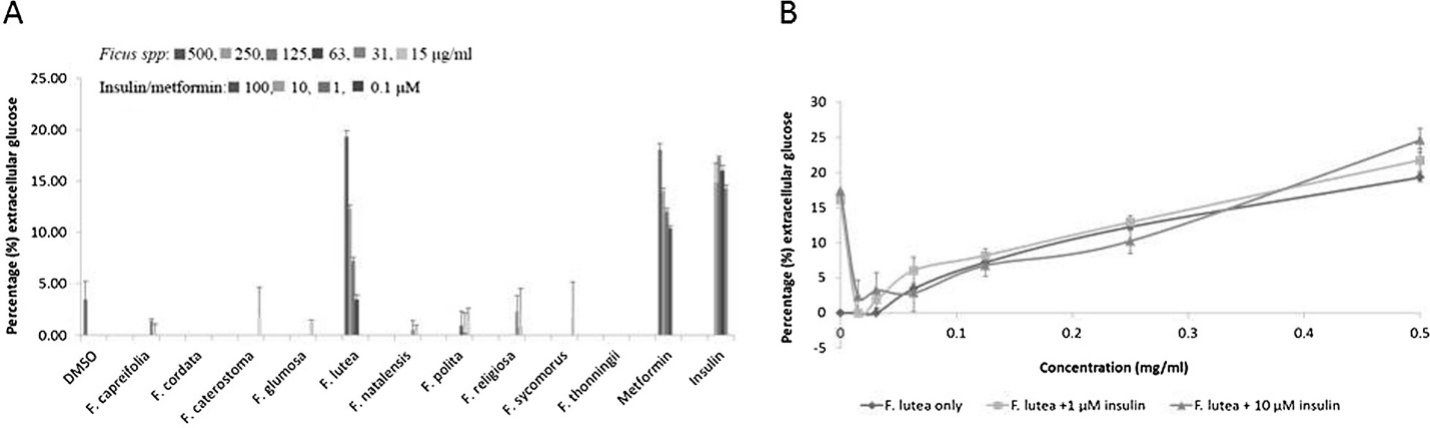
**Extracellular glucose concentration of H-4-11-E rat liver cells (A) expressed as percentage of untreated control cells ± standard error of mean, n = 9 exposed to the acetone extracts of the ten** ***Ficus*** **species, insulin or metformin and (B) expressed as percentage of untreated control cells ± standard error of mean, n = 9 exposed to insulin at 1 or 10 μM, in combination with** ***F. lutea*** **at various concentrations.**

The effect of the acetone extracts of the ten *Ficus* species on extracellular glucose concentration of H-4-II-E liver cells is presented in Figure 2A. Only *F. lutea* extract significantly (*p*<001) decreased extracellular glucose concentration [12.9 ± 0.6 μg/ml (19.3 ± 0.6%)] at the highest concentration (500 μg/ml) into H-4-II-E liver cells. The solvent control (DMSO) had minimal glucose uptake activity of 2.3 ± 1.2 μg/ml (3.4 ± 1.8%), while metformin and insulin significantly (*p*<0.001) decreased extracellular glucose concentrations with the greatest decrease being 12.0 ± 0.4 μg/ml (18.1 ± 0.6%) and 11.5 ± 0.1 μg/ml (17.3 ± 0.1%) respectively. With only the *F. lutea* extract being effective, the activity of the *F. lutea* extract in the presence of insulin was investigated (Figure 2B). *Ficus lutea*, at the highest concentration (500 μg/ml) in the presence of insulin (concentrations of 1 μM and 10 μM, decreased the extracellular glucose concentrations of H-4-II-E liver cells significantly (*p*<0.05) [14.5 ± 1.2 μg/ml (21.8 ± 1.6%)] and (*p*<0.001) [16.4 ± 1.3 μg/ml (24.6 ± 1.7%)] at both insulin concentrations compared to the extract and insulin alone. A marginal decrease was seen for the lower doses of *F. lutea* with insulin present in comparison to its absence. As above, insulin at both the tested concentrations did not have its maximum effect of 10.7 ± 0.3 μg/ml) 16.0 ± 0.5% and 11.5 ± 0.1 μg/ml (17.3 ± 0.1%), respectively when combined with the *F. lutea* extract.

#### Glucose uptake in 3T3-L1 pre-adipocytes

**Figure 3:**
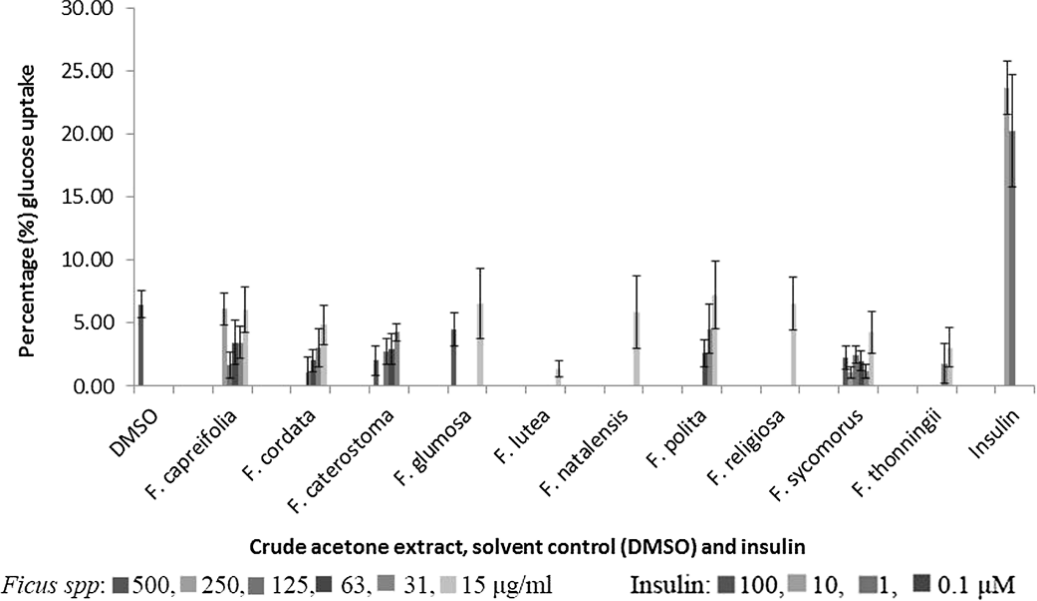
**Glucose uptake in 3T3-L1 pre-adipocytes (expressed as percentage of untreated control cells ± standard error of mean, n = 9) exposed to the acetone extracts of the ten** ***Ficus*** **species or insulin.**

No extract of any of the *Ficus* species enhanced glucose uptake in the 3T3-L1 pre-adipocytes even at the highest concentration of 500 μg/ml. Insulin, the positive control, significantly (*p*<0.001) enhanced glucose uptake in 3T3-L1 pre-adipocytes with the highest glucose uptake of 15.8 ± 1.8 μg/ml (23.7 ± 2.1%) at the concentration 10 μM while the uptake in the DMSO treated cells was 4.3 ± 0.9 μg/ml (6.5 ± 1.1%) (Figure 3).

### Glucose uptake activity in primary cell culture

**Figure 4:**
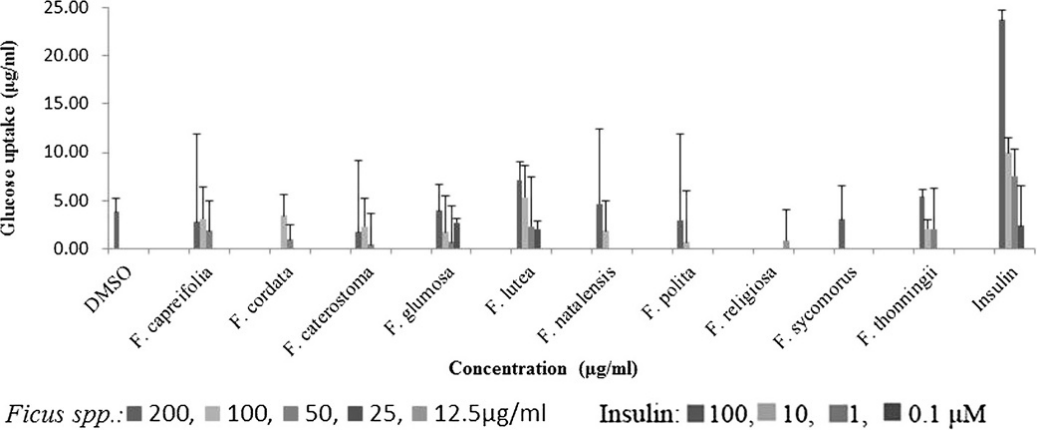
**Glucose uptake (μg/ml) of rat abdominal primary muscle cultures (± standard error of mean, n = 9) exposed to the acetone extracts of the ten** ***Ficus*** **species and insulin in the present of 1 mM glucose.**

**Figure 5:**
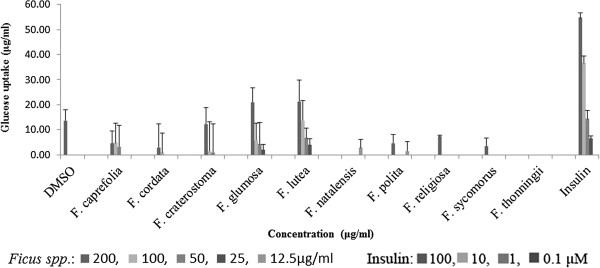
**Glucose uptake (μg/ml) of rat epididymal primary fat cell cultures (± standard error of mean, n = 9) exposed to the acetone extracts of the ten Ficus species and insulin in the present of 1 mM glucose.**

The effect of the acetone extracts of the ten *Ficus* species on glucose uptake in primary rat abdominal muscle cultures at 1 mM glucose is presented in Figure 4. *Ficus lutea* extract at the concentration of 200 μg/ml induced the highest [7.2 ± 1.3 μg/ml (10.8 ± 1.8%)] glucose uptake into muscle cultures but was not significantly different (*p*<0.05) from glucose uptake induced by the extracts of *F. thonningii* [5.4 ± 0.4 μg/ml (8.1 ± 0.7%)], *F. natalensis* [4.7 ± 6.5 μg/ml (7.0 ± 7.7%)] and *F. glumosa* [3.9 ± 2.4 μg/ml (5.9 ± 2.7%)] at the same concentration and in comparison to the DMSO solvent control [3.8 ± 0.9 μg/ml (5.7 ± 1.4%)]. This uptake was only 33% of that induced by insulin [23.8 ± 1.1 μg/ml (35.7 ± 1.0%)] at 100 μM. The effect of the acetone extracts of the ten *Ficus* species on glucose uptake in rat epididymal fat cells at 1 mM glucose is presented in Figure 5. Only the extracts of *F. lutea* and *F. glumosa* significantly (*p*<0.001) induced glucose uptake in the primary fat cell cultures of 21.3 ± 6.2 μg/ml (32.0 ± 8.4%) and 21.0 ± 4.8 μg/ml (31.6 ± 5.7%) respectively at 200 μg/ml. As was the case for the abdominal muscle, the solvent control (DMSO) also had some activity [13.7 ± 3.9 μg/ml (20.6 ± 4.2%)]. Insulin was most effective with uptake of 54.8 ± 1.1 μg/ml (82.2 ± 2.0%) at 100 μM.

#### Insulin secretion in RIN-m5F pancreatic cells

**Figure 6:**
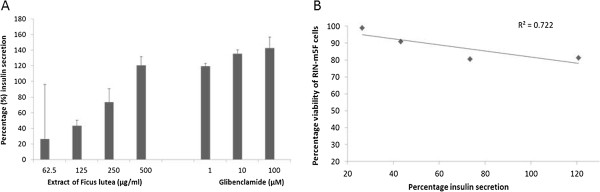
**Insulin secreted by RIN-m5F pancreatic cells (A) expressed as percentage of untreated control cells ± standard error of mean, n = 6 exposed to the acetone extract of** ***F. lutea*** **and glibenclamide (positive control) in glucose free medium and (B) the correlation between percentage cell viability of RIN-m5F pancreatic β-cells and percentage insulin secretion by the acetone extract of** ***F. lutea*** **.**

With only *F. lutea* extract having a good activity in the above assays, the acetone extract of *F. lutea* was also evaluated for its insulin secretory activity in RIN-m5F pancreatic β-cells (Figure 6A). A concentration dependent increase in insulin secretion was present for *F. lutea*, with significant (*p*<0.001) increase from 6.3 ± 0.2 μg/L to 11.1 ± 0.3 μg/L (26.4 ± 6.4% to 120.8 ± 13.7%) from concentrations of 62.5 μg/ml to 500 μg/ml. Glibenclamide also significantly (*p*<0.001) increased insulin secretion in a concentration dependent manner. An inverse linear correlation was present between cell viability and insulin release for the *F. lutea* extract (Figure 6B).

#### Structure elucidation of the isolated compound

**Figure 7:**
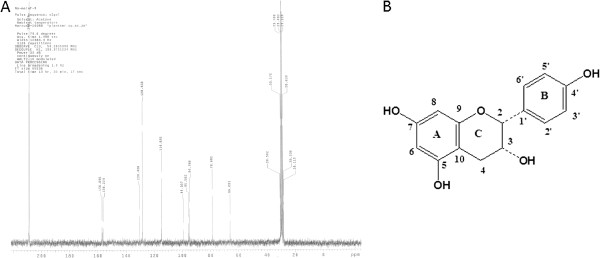
^**13**^ **C-NMR (125 MHz, CDCI**_**3**_ **) Spectrum** **(A) and the structure of epiafzelechin (B) isolated from the leaf acetone extract of** ***F. lutea.***

The ^13^C NMR and DEPT (Distortionless Enhancement by Polarisation Transfer) spectra exhibited signals at *δ* 78.7, 66.1 and 28.1 ppm corresponding to carbons C-2, C-3 and C-4 characteristic for a flavan-3-ol skeleton. This was confirmed by the presence of important signals on its ^1^H NMR spectrum at *δ* 4.87 (*brs*, H-2), 4.15 (*m*, H-3), 2.80 (*dd*, 4.4, 16.8 Hz, H-4_a_) and 2.62 (*dd*, 3.4, 16.6 Hz, H-4_b_) due to protons at positions C-2, C-3 and C-4, respectively. Two sets of aromatic protons were observed on its ^1^H NMR spectrum: the first set appeared as two doublets at *δ* 5.98 (*d*, 2.2 Hz, H-8) and 5.87 (d, 2.2 Hz, H-6) due to the ring-A while the second one appeared as AA'BB' system at *δ* 7.28 (*d*, 8.4 Hz, H-2'/H-6') and 6.76 (*d*, 8.4 Hz, H-3'/H-5') corresponding to B-ring. Furthermore, the ^1^H NMR spectrum also displayed three downfield broad signals between 9.5 and 8.9 ppm assignable to three hydroxyl groups at C-4', C-7 and C-5. The broad singlet multiplicity of proton H-2 was indicative to this proton to be in *cis* configuration with proton H-3. Moreover, the ^13^C NMR spectrum exhibited signals at *δ* 156.9, 156.2, 130.4 and 99.0 ppm corresponding to carbons C-7/C-4', C-5/9, C-1' and C-10, respectively. All these data were in agreement with those published for epiafzelechin (Figure 7) [35]. This compound which was previously reported from *Ficus cordata* [36] is isolated here for the first time from this species.

#### Effect of epiafzelechin on glucose uptake in C2C12 muscle cells

**Figure 8:**
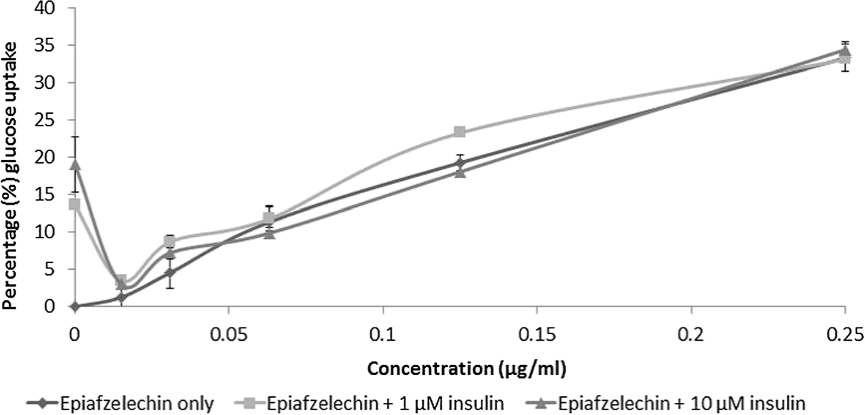
**Glucose uptake in C2C12 muscle cells (expressed as percentage of untreated control cells ± standard error of mean, n = 9) exposed to insulin at 1 or 10 μM, in combination with epiafzelechin.**

The effect of epiafzelechin on glucose uptake by the muscle cells, in the presence of insulin was subsequently evaluated. The uptake of glucose by C2C12 cells treated with epiafzelechin at different concentrations (15 μg/ml – 250 μg/ml) in medium containing two different concentrations of insulin (1 μM and 10 μM) is presented in Figure 8. The insulin-mediated glucose uptake of C2C12 exposed to epiafzelechin at different insulin concentrations of 1 μM and 10 μM was 22.1 ± 0.2 μg/ml (33.2 ± 0.5%) and 23.0 ± 0.7 μg/ml (34.5 ± 1.1%) respectively compared to epiafzelechin alone [22.2 ± 0.9 μg/ml (33.4 ± 1.8%)] at the highest epiafzelechin concentration (250 μg/ml). As seen with the acetone extract, epiafzelechin decreased the effectivity of insulin at the lower concentrations.

#### Effect of epiafzelechin on extracellular glucose concentration of H-4-11-E liver cells

**Figure 9:**
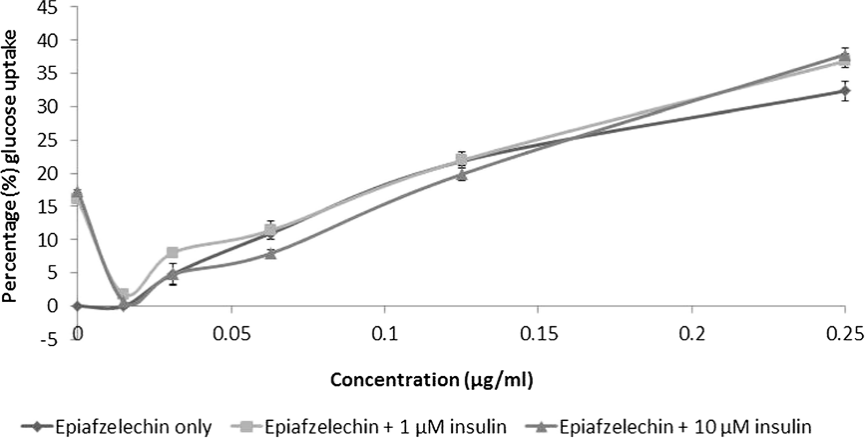
**Extracellular glucose concentration of H-4-11-E liver cells (expressed as percentage of untreated control cells ± standard error of mean, n = 9) exposed to insulin at 1 or 10 μM, in combination with epiafzelechin.**

The effect of epiafzelechin on glucose uptake by the H-4-II-E liver cells, in the presence of insulin was evaluated. The uptake of glucose by C2C12 cells treated with epiafzelechin at different concentrations (15 μg/ml – 250 μg/ml) in medium containing two different concentrations of insulin (1 μM and 10 μM) is presented in Figure 9. The insulin-mediated glucose uptake in H-4-II-E liver cells exposed to epiafzelechin at different insulin concentrations of 1 μM and 10 μM was 24.6 ± 1.1 μg/ml (36.9 ± 1.0%) and 25.3 ± 0.7 μg/ml (37.9 ± 0.9%) respectively when compared to epiafzelechin alone [21.6 ± 1.0 μg/ml (32.4 ± 1.5%)] at the highest concentration (250 μg/ml). As seen with the acetone extract, epiafzelechin decreased the effectivity of insulin at the lower concentrations.

#### Effect of epiafzelechin on insulin secretion in RIN-m5F pancreatic β-cells

**Figure 10:**
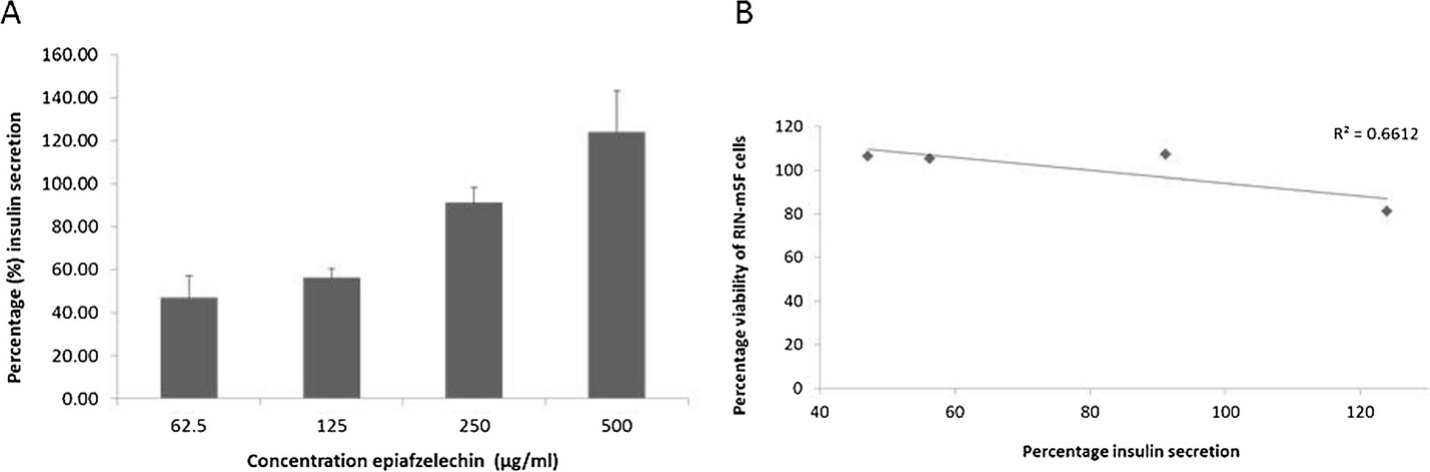
**Insulin secreted by RIN-m5F pancreatic cells (A) expressed as percentage of untreated control cells ± standard error of mean, n = 6 exposed to epiafzelechin in glucose free medium and (B) the correlation between percentage cell viability of RIN-m5F pancreatic β-cells and percentage insulin secretion by epiafzelechin.**

Epiafzelechin was evaluated at different concentrations (62.5 μg/ml – 500 μg/ml) for its ability to stimulate insulin secretion in RIN-m5F pancreatic β-cells and was compared with the untreated control cells. The RIN-m5F pancreatic cells exposed to the epiafzelechin resulted in a dose related increase in insulin secretion (Figure 10A). The insulin secreted significantly (*p*<0.001) increased from 7.2 ± 0.21 μg/L (47.1 ± 10.2%) at the concentration of 62.5 μg/ml to 11.2 ± 0.61 μg/L (123.9 ± 19.2%) at the concentration of 500 μg/ml. The correlation coefficient between the viability of RIN-m5F pancreatic β-cells and insulin secretion by the ethyl acetate fraction of the extract of *F. lutea* R^2^ was 0.66 (Figure 10B).

## Discussion

Of the ten *Ficus* spp. evaluated, only *F. lutea* appeared to have the ability to modulate glucose metabolism. When the glucose uptake ability of the plant extract was compared to insulin in the primary muscle cells, the extract was 33% as effective. In the hepatoma cell line, the *F. lutea* plant extract was as effective as metformin in decreasing extracellular glucose concentration, by approximately 20%. In the pancreatic secretory assay, the *F. lutea* extract was 4 times greater in its secretory activity than commercial glibenclamide. As a result it would appear that only *F. lutea* has merit in the management of type II diabetes.

The other aim of this study was to ascertain the mechanism by which any of the plants could decreases extracellular glucose concentration. This was facilitated through the use of cell types with different transporter expression criteria for the glucose transport receptors (GLUT), insulin secretion and/or gluconeogenesis. The 3T3-L1 pre-adipocytes predominantly express the GLUT1 glucose transporter over GLUT4 [37]; the differentiated C2C12 myocytes and the primary cells the GLUT4 transporter and the H-4-II-E hepatoma cells the GLUT2 glucose transporter [38]. While the 3T3-L1 pre-adipocytes can be transformed into adipocytes that express the higher quantities of GLUT4 transporter (5 fold higher), this transformation step was not attempted for this study as we were only interested in using the pre-adipocytes as the control in comparison to the other GLUT4 expressing cell lines [39–42]. The hepatoma cell line was also used to study the influence of the extract on gluconeogenesis [43], while the pancreatic cells were used to evaluated insulin secretory activity.

Based upon the poor glucose uptake by the non-differentiated 3T3-L1 pre-adipocytes and the significantly increasing glucose uptake in the primary muscle cells, primary fat cells, C2C12 muscle and H-4-II-E liver cells, we suspect that the *F. lutea* extract had its effect by increasing the activity of cell surface glucose transporters either directly or indirectly. With the 3T3-L1 pre-adipocytes being poor in their glucose transporting ability due to low levels of expression of the GLUT4 receptor, this transporter must be considered a possible target site [44]. Furthermore the plant extract did not have the ability to induce differentiation in the pre-adipocytes, indicating the absence of glucocortisteroid capacity. However, with the hepatoma cell lines also showing a decrease in extracellular glucose concentration, decreased gluconeogenesis (with effects being similar to metformin) and/or increased GLUT2 transporter activity are other potential mechanisms. While it is possible that the *F. lutea* plant extract is effective at multiple pathways (GLUT4, GLUT2 and gluconeogenesis), a more plausible explanation is that the extract stimulates the insulin receptor, which on activation would increase GLUT4/2 transporter activity and/or decrease hepatic gluconeogenesis depending on the cell type. However, for the mechanism of action to be fully characterised, further studies are required. One such method could involve evaluating the effects of the *F. lutea* acetone extract on glucose concentrations and/or tyrosine kinase activities, in the presence of an antagonist of the insulin receptor. In studies using the S961 peptide, Sprague Dawley rats showed both a hyperglycaemia and hyperinsulinaemia response when treated with this peptide [45]. If the effect seen by *F. lutea* acetone extract is indeed mediated via the insulin receptor, one would expect a competitive reduction in the effect achievable or further downstream effects with increasing concentrations of the antagonist added.

To further evaluate whether the effect of the *F. lutea* extract was being mediated via the insulin tyrosine kinase receptor or related pathways, the effect of insulin alone was compared to that with concurrent *F. lutea*, or to *F. lutea* alone. The extract of *F. lutea* failed to further enhance the effect of insulin. For both tissue types an odd profile was attained, as the lower dose of *F. lutea* appeared to inhibit the response to insulin, while the high dose appeared to be marginally superior to insulin alone, in a manner we consider to be mixed agonistic/antagonistic activity. The latter response could also explain why the plant extract achieved only a third of the effect of insulin in the primary muscle tissue as it is in essence a partial agonist when acting alone. This result was also similar to that observed with resveratrol, a wine polyphenol which, when used in the absence of insulin, enhanced muscular uptake of glucose, but when added simultaneously to insulin led to a time dependent diminishing of glucose uptake in C1C12 muscle cells [46].

In the final step in elucidating the mechanism of action of *F. lutea,* the influence of the plant extract on insulin secretion was investigated. While not a completely effective means of treating diabetes, the enhanced release of insulin early on in the disease pathogenesis has been known to ameliorate hyperglycaemia. The extracts of *F. lutea* stimulated insulin secretion (9 fold) in the RIN-m5F pancreatic cells in comparison to glibenclamide (5 fold). While our result thus suggests that the extract of *F. lutea* could possess insulin secretagogue properties, correlation analysis shows an inverse correlation between cell viability and insulin release. This may suggest that the insulin secretory effect seen was an artefact, more likely as a result of the high concentrations inducing cell lyses as opposed to a physiological effect.

One of the compounds we isolated from *F. lutea* acetone extract was identified as epiafzelechin. As far as it could be established, this is the first study to demonstrate that epiafzelechin has hypoglycaemic activity, as previously shown for other polyphenolic compounds using *in vitro* muscle and fat cultures [47]. In this study, epiafzelechin enhanced insulin secretory activity of RIN-m5F pancreatic β-cells, albeit with correlation with cell toxicity. It also enhanced glucose uptake in C2C12 muscle cells and decreased extracellular H-4-II-E liver cells glucose concentration in excess of that of the crude extract, most likely due to the same agonistic/antagonistic insulin-mimetic mode of action. When insulin and epiafzelechin were added simultaneously to the C2C12 muscle and H-4-II-E liver cells, there was no significant (*p*<0.05) change in activity to that in the absence of insulin at the highest concentration. These results are similar to those observed by Ueda *et al.* [48], where epigallocatechin gallate stimulated a dose dependent increase in glucose uptake of L6 muscle cells with no synergism being present with insulin.

## Conclusion

This study investigated the potential antidiabetic activity of ten *Ficus* species, focussing on glucose uptake in muscle, fat and liver cells; insulin secretion and safety through cytotoxicity assay. From these traditionally used plant species, only *F. lutea* possessed substantial *in vitro* activity related to glucose metabolism. When glucose uptake is compared, the plant species was only 33% as effective as insulin. In comparison to metformin, the plant extract was as effective in inhibiting gluconeogenesis. The plant also appears to lack direct insulin secretory activity. Based on the effect produced in the various cell types, *F. lutea* also appears to be a partial agonist/antagonist of the insulin cell membrane receptor. However, for the true effect of *F. lutea* to be established, *in vivo* oral rodent efficacy studies in a disease model will be required. The latter is required to determine the biophasic availability of the extract and/or its active constituents and its relationship to efficacy following oral administration. More importantly the interaction of the drug with the insulin receptor in the diseased state will also require elucidation.
